# Caloric restriction induces anabolic resistance to resistance exercise

**DOI:** 10.1007/s00421-020-04354-0

**Published:** 2020-03-31

**Authors:** Chaise Murphy, Karsten Koehler

**Affiliations:** 1 Department of Nutrition and Health Sciences, University of Nebraska-Lincoln, Lincoln, NE USA; 2 Department of Sport and Health Sciences, Technical University of Munich, Uptown München-Campus D, Georg-Brauchle-Ring 60/62, 80992 München, Germany

**Keywords:** Energy deficit, Energy availability, Weightlifting, Strength training, Growth hormone, Sclerostin

## Abstract

**Purpose:**

Weight loss can result in the loss of muscle mass and bone mineral density. Resistance exercise is commonly prescribed to attenuate these effects. However, the anabolic endocrine response to resistance exercise during caloric restriction has not been characterized.

**Methods:**

Participants underwent 3-day conditions of caloric restriction (15 kcal kg FFM^−1^) with post-exercise carbohydrate (CRC) and with post-exercise protein (CRP), and an energy balance control (40 kcal kg FFM^−1^) with post-exercise carbohydrate (CON). Serial blood draws were taken following five sets of five repetitions of the barbell back squat exercise on day 3 of each condition.

**Results:**

In CRC and CRP, respectively, growth hormone peaked at 2.6 ± 0.4 and 2.5 ± 0.9 times the peak concentrations observed during CON. Despite this, insulin-like growth factor-1 concentrations declined 18.3 ± 3.4% in CRC and 27.2 ± 3.8% in CRP, which was greater than the 7.6 ± 3.6% decline in CON, over the subsequent 24 h. Sclerostin increased over the first 2 days of each intervention by 19.2 ± 5.6% in CRC, 21.8 ± 6.2% in CRP and 13.4 ± 5.9% in CON, but following the resistance exercise bout, these increases were attenuated and no longer significant.

**Conclusion:**

During caloric restriction, there is considerable endocrine anabolic resistance to a single bout of resistance exercise which persists in the presence of post-exercise whey protein supplementation. Alternative strategies to restore the sensitivity of insulin-like growth factor-1 to growth hormone need to be explored.

**Electronic supplementary material:**

The online version of this article (10.1007/s00421-020-04354-0) contains supplementary material, which is available to authorized users.

## Introduction

While weight loss is necessary to combat obesity and its associated comorbidities, it may negatively impact both the muscular (Weinheimer et al. 2010) and skeletal (Ensrud et al. 2018) systems. Weight loss consistently reduces muscle protein synthesis (Hector et al. 2018; Pasiakos et al. 2013) and has been found to increase muscle protein breakdown (Carbone et al. 2014). These changes parallel the suppression of bone formation (Ihle and Loucks 2004) and elevation of bone resorption (Ihle and Loucks 2004) during weight loss. Thus, exercise is often recommended to attenuate the insults of caloric restriction to the musculoskeletal system (Weinheimer et al. 2010). Though both aerobic and resistance exercise have been shown to preserve lean mass (Weiss et al. 2017; Sardeli et al. 2018) and bone mineral density (Armamento-Villareal et al. 2012; Villareal et al. 2006) during calorie-restricted weight loss, some evidence suggests that resistance training may be superior for preserving lean mass (Clark 2015; Villareal et al. 2017) and bone mineral density (Beavers et al. 2017; Armamento-Villareal et al. 2019), potentially due to the larger anabolic endocrine response generated by higher intensity exercise protocols (Wahl et al. 2013).

However, the response of anabolic hormones to resistance exercise may be altered under caloric restriction. At energy balance, growth hormone (GH) secretion from the anterior pituitary stimulates insulin-like growth factor-1 (IGF-1) production, primarily in the liver (Vottero et al. 2013). In turn, the resulting increase in IGF-1 provides negative feedback to the hypothalamus and anterior pituitary, reducing the production of GH releasing hormone and GH, respectively (Vottero et al. 2013). Previous research has demonstrated caloric restriction disrupts the GH:IGF-1 axis, such that increasing GH secretion does not stimulate IGF-1 production and, in turn, there is no subsequent negative feedback to reduce GH production (Fazeli and Klibanski 2014). These alterations occur in a dose-dependent fashion, such that higher levels of caloric restriction produce greater increases in GH and reductions in IGF-1 compared to lower levels of caloric restriction and energy balance (Loucks and Thuma 2003). This dysregulated pairing of increased GH and decreased IGF-1 has been termed growth hormone resistance (Fazeli and Klibanski 2014) and represents a specific form of anabolic resistance. However, whether this dysregulation persists in the face of a potent anabolic stimulus, such as resistance training, has not been investigated.

The responses of systemic anabolic factors, such as GH and IGF-1, warrant consideration as both hormones play significant roles in the development of the skeletal system (Tritos and Klibanski 2016). The reduction in IGF-1 during caloric restriction, specifically, has been associated with bone loss (De Souza and Williams 2005), and bone lost during weight loss is not easily restored (Villalon et al. 2011). Previous studies have used short-term caloric restriction to induce substantial changes in markers of bone turnover (Papageorgiou et al. 2017; Loucks and Thuma 2003). Changes in markers of bone turnover appear before noticeable changes in bone mineral density can be observed (Fujimura et al. 1997), but have been shown to parallel changes in bone mineral density in long-term studies (Villareal et al. 2016). Therefore, markers of bone turnover can serve as reliable indicators of the shift in bone metabolism during short-term interventions. Investigating the short-term effects of resistance exercise on markers of bone turnover during caloric restriction is an important first step towards refining diet and exercise guidelines to preserve bone during weight loss. By understanding whether the response is suppressed by caloric restriction, we can devise strategies to overcome this suppression in an acute setting and, if applied repeatedly, attenuate the loss of bone mineral density.

To maximize the potency of the anabolic response to resistance exercise, dietary protein is often manipulated in concert with resistance exercise. Six months of twice daily protein supplementation in combination with resistance training has been reported to increase IGF-1 concentrations at energy balance (Ballard et al. 2005). During caloric restriction, a high-protein diet in combination with resistance training has been shown to preserve muscle protein synthesis rates nearer to those observed at energy balance compared to a low-protein diet (Hector et al. 2018) and preserve, or even accrue, lean mass (Longland et al. 2016). Supplementation of whey protein after a bout of resistance exercise has been shown to elevate muscle protein synthesis above resting levels at energy balance (Areta et al. 2014), while resistance training alone has been shown to match, but not exceed, those observed at energy balance (Murphy et al. 2015).

Thus, to inform the development of strategies for maximizing the anabolic response to a bout of resistance exercise during caloric restriction, we first sought to measure the impact of short-term caloric restriction on the anabolic response to a bout of resistance exercise. Additionally, we quantified the impact of a single resistance exercise bout under conditions of caloric restriction on markers of bone turnover, namely sclerostin and N-terminal propeptide of type-1 collagen (P1NP), which has been shown to correlate with IGF-1 (Niemann et al. 2013). Finally, we wanted to test the impact of post-exercise protein supplementation on the anabolic response to resistance exercise in the calorie-restricted state. We hypothesized that GH would be significantly elevated and IGF-1 would be significantly suppressed following resistance exercise in the calorie-restricted state compared to energy balance, indicating the development of anabolic resistance. We further hypothesized that a bout of resistance exercise would elevate bone formation, measured through P1NP, and reduce sclerostin, a measure of anti-bone formation, even under caloric restriction. Finally, we hypothesized that post-exercise protein supplementation would attenuate the suppression of IGF-1 following a bout of resistance exercise in the calorie-restricted state.

## Methods

### Study design

The present randomized, single-blind repeated-measures crossover trial consisted of three 3-day conditions. Two conditions restricted energy intake to 15 kcal kg FFM^−1^ (CR), while the third provided 40 kcal kg FFM^−1^, operationally defined as the control condition (CON). These levels of energy availability have been previously shown to induce weight loss and maintain weight, respectively, during a similar short-term intervention (Koehler et al. 2016). All conditions provided participants 1.2 g kg body weight (BW)^−1^ protein, which has previously been shown to maintain lean mass in combination with resistance training during caloric restriction (Longland et al. 2016). Following a resistance exercise bout on day 3 of each condition, participants consumed a post-exercise protein beverage during one CR condition or a post-exercise carbohydrate beverage during the other CR condition and CON. Participants underwent conditions in a random order and completed a washout period of at least 2 weeks between conditions during which they resumed habitual exercise and dietary practices. With one exception, all participants completed all conditions within 8 weeks of the same school semester. The study was approved by the University of Nebraska—Lincoln’s Institutional Review Board and registered at www.clinicaltrials.gov (NCT03600311).

### Participants

Participants were recruited from campus and other local recreation sites via flyers, emails to campus sports clubs, and social media posts between August 1st, 2018 and May 1st, 2019. Participants were height- and weight-stable (< 0.25 inches and < 2.5 kg change in last 6 months) men and women between 19 and 30 years old with a lean body fat percentage (< 20% men, < 30% women) for their age (Borrud et al. 2010). Participants were currently active recreational weightlifters with at least 3 years of resistance training experience, which we assessed with an online questionnaire. We selected young, lean, trained participants for their larger anabolic response to exercise (Häkkinen et al. 1998; Thomas et al. 2011; Rubin et al. 2005). Young participants also have larger anabolic responses to protein intake (Moore et al. 2015) compared to older adults and lean participants lose greater amounts of lean mass during weight loss (Forbes 2000). All of these factors served to maximize our effect sizes. Recruiting trained participants ensured that participants would be able to safely complete a high-intensity bout of resistance exercise under fasted, calorie-restricted conditions. Compliance to these inclusion criteria was confirmed during an initial screening visit to the laboratory after the informed consent was signed.

### Preliminary testing

During the preliminary testing, participants had their height and weight taken by an electronic stadiometer (SECA, Germany) and their body composition was estimated by bioimpedance analysis (BIA; Quadscan 4000, BodyStat, UK). Each participant performed a familiarization session in the power rack used for the barbell back squat exercise during each condition. Briefly, participants were first provided with the option of performing a self-selected warm-up from available equipment, including a treadmill, cycle ergometer, and foam roller. Participants then completed between 2 and 5 warm-up sets of the barbell back squat exercise. Once participants indicated that they were warmed-up, they selected a weight with which they knew they could complete five repetitions. Following the set, participants provided the number of repetitions in reserve (RIR) they felt they had on the previous set. Participants then increased the weight and attempted another set until they indicated ≤ 1 RIR or failed to complete five repetitions. All participants satisfied one of these criteria within three working sets. Rest intervals between sets were not controlled during preliminary testing.

### Diet preparation

Participants were provided all food consumed during each 3-day condition. Diets consisted of an individually tailored combination of clinical products (Ensure Plus; 4.57 g protein · 100 kcal^−1^ and Ensure High Protein; 10 g protein · 100 kcal^−1^, Abbott Nutrition, USA), maltodextrin (Tate & Lyle, UK), and whey protein isolate (Isopure, USA). Participants were allowed to consume their meals in 3–4 sittings throughout the day and were asked to record their meal timings. Blinding was achieved by matching the total volume between conditions via dilutions with water. During the conditions, participants were permitted to consume water ad libitum, but no other products.

**Fig. 1:**
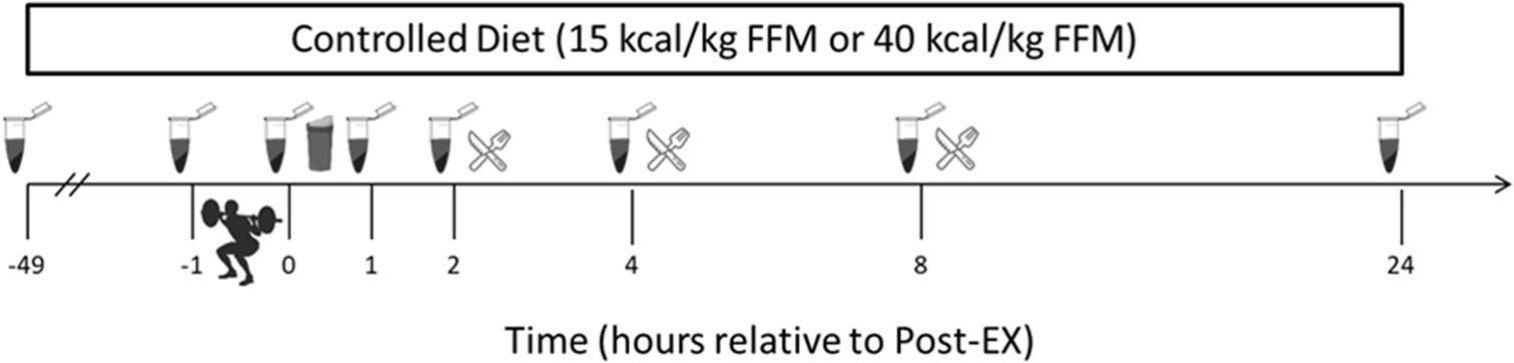
Timeline of blood draws, resistance exercise bout, post-exercise protein or carbohydrate supplementation and day-3 meals during each 3-day condition

Following their resistance exercise bout on day 3, participants received isocaloric post-exercise beverages consisting of 30 g whey protein isolate [CR with protein (CRP)] or maltodextrin [CON and CR with carbohydrate (CRC)] dissolved in 400 mL water. These beverages were consumed in addition to the provided 15 or 40 kcal · kg FFM^−1^ and 1.2 g · kg BW^−1^ protein. Participants were blinded to which beverage they received through a flavored water enhancer. Meals on day 3 were consumed at standardized times relative to blood draws to minimize interference with the exercise response (Fig. 1).

### Supplementation

To mitigate differences in calcium and vitamin D consumption, we supplemented participant intake of these micronutrients throughout the entire study, including washout periods. Calcium and vitamin D provided by each condition were supplemented to make up the difference from the maximal amount provided during one condition. Supplementation of calcium during washout periods was calculated by subtracting habitual calcium intake from the maximal value provided by any condition. Habitual calcium intake through the diet was determined using the Brief Calcium Assessment Tool (Yang et al. 2010). Vitamin D was supplemented at the maximal amount provided by any condition. Participants were provided all supplements in pill boxes spacing them into 1–3 doses per day depending on number of supplements needing to be consumed.

### Resistance exercise bout

On day 3 of each condition, participants reported to the laboratory between 0700 and 0900 h following an overnight fast (≥ 10 h) to perform five sets of five repetitions (5 × 5) of the barbell back squat exercise. All visits for a participant occurred within 0.5 h of the same time each morning. Participants utilized the same warm-up procedures from their preliminary testing visit before beginning their first set of the 5 × 5 with the heaviest weight at which they successfully completed five repetitions with at least 1 RIR during the preliminary testing visit. After the first set, participants provided their RIR and the weight was adjusted according to a standardized system. Participants who indicated 0 RIR or did not complete their set decreased the weight on the bar. When participants indicated 1 or 2 RIR, the weight on the bar stayed the same in the next set. If participants indicated 3 or more RIR, the weight on the bar increased for the next set. Between working sets, participants were required to rest for at least 2 min and could not rest longer than 5 min. A large rest range was permitted to ensure that participants were able to recover between sets in the manner which they habitually trained.

Participants were not allowed to exercise 24 h prior to or during each 3-day condition outside of their 5 × 5 exercise bout. Strenuous physical activity was also discouraged. Compliance with these procedures was assessed via a waist-worn accelerometer (ActiLife G3TX + , ActiGraph, USA).

### Body weight and composition

Before and after each 3-day condition, participants reported to the laboratory following an overnight fast of at least 10 h where body weight was measured and body composition was assessed via Dual-Energy X-ray Absorptiometry scans (iDXA, GE Healthcare, USA). We assessed hydration status by measuring the specific gravity of each morning urine (Armstrong et al. 2010).

### Blood collection and serum assays

Assays were performed on fasted blood samples collected in the morning of days 1, 3, and 4 of each condition. Additional samples were obtained serially 0-, 1-, 2-, 4-, and 8-h post-exercise on day 3 of each condition (Fig. 1). All blood samples collected throughout the study were stored as serum aliquots at − 80 °C until analysis. Commercially available assays were used to measure serum concentrations of IGF-1 [R&D Systems], GH [R&D Systems], P1NP [ABClonal], and sclerostin [Biomedica]. Our intraassay variabilities for each assay were 2.44% (IGF-1, sensitivity: 0.056 ng/mL), 4.82% (GH, sensitivity: 7.18 pg/mL), 9.23% (P1NP, sensitivity: 0.91 ng/mL), and 8.31% (sclerostin, sensitivity: 72 pg/mL).

### Calculations

Prior to data analysis, data were examined for outliers, which were removed from the data set prior to proceeding with analysis. Missing body composition data from one CR condition scan in one participant as a result of a machine malfunction were imputed using the participants’ other CR condition. This decision was made due to the similarity in pre-condition mass and compartment mass between the two CR conditions (< 0.1 kg difference for all measurements) and the assumption that weight loss, and the composition of weight loss, would not differ between the two isocaloric CR conditions. To minimize the impact of sex differences in GH secretion (Luk et al. 2015), we normalized all time points of GH collection to the peak in the CON condition for each participant. Area under the curve (AUC) was calculated for GH as the area above 0 using the trapezoidal method. AUC for IGF-1 was calculated as the area below the day 3 Pre-Exercise blood draw in the same manner. Volume of exercise bouts was calculated as the product of weight lifted in kg relative to body weight in kg times the number of reps completed at that weight. Changes between time points were expressed in the original units for body composition and percentages for IGF-1 and markers of bone turnover. Concentrations from serial time points were reported in the original units for IGF-1 due to the similarity in the initial concentrations.

### Statistical analyses

We first used one-sided *t* tests to determine if changes in hormone concentrations or body composition between time points in each condition were significantly different from 0 in hypothesized directions. If changes were significantly different from 0 and inspection of the data suggested that group differences may exist, planned pairwise comparisons, a type of contrast, were used to test for group differences between CR and CON or CRC and CRP. To test whether the anabolic response to resistance exercise was altered during CR compared to energy balance, we applied a contrast to compare the AUC responses of GH and IGF-1 between CR and CON. To test whether resistance exercise was able to rescue changes in markers of bone turnover in CR, we compared the changes observed between day 1 and day 3 against those seen between day 1 and day 4 in each condition. Finally, to test whether post-exercise protein supplementation could rescue the blunted IGF-1 response to exercise, we applied a contrast to compare the AUC responses of IGF-1 between CRC and CRP. Differences in urine-specific gravity were assessed with an omnibus *F* test. Additionally, we reported Cohen’s *d*, or the difference in group means divided by the pooled standard deviation, as effect sizes (Cohen 1988). Sample size was determined based on the literature reporting changes in IGF-1 following the reduction in energy availability to 10 kcal kg FFM^−1^ day^−1^ or 20 kcal kg FFM^−1^ day^−1^ for 5 days (Loucks and Thuma 2003). Based on these data, the expected *d* was between 1.2 and 1.5, and a sample size of *n* = 6 was sufficient to detect between-group differences of 1.2 with a power of 0.80. All statistical analysis was performed using R (R Core Team, Version 3.6). Unless otherwise stated, all data in text and figures are reported as mean ± standard error of the mean. We defined statistical significance as *p* < 0.05.

## Results

### Participant characteristics and compliance

Of the 15 participants who started an intervention, ten completed at least one condition and eight participants completed all three conditions. One participant was excluded retrospectively due to noncompliance with study procedures [Figure S1]. At baseline, the seven participants (five men and two women) included in the present analysis were 22 ± 2 years of age and weighed 79.4 ± 7.3 kg with 18.5 ± 2.7% body fat. They had 6 ± 1 years of resistance training experience and successfully completed five repetitions at 1.4 ± 0.1 times their body weight during preliminary testing.

### Changes in body weight and composition

Participants lost weight in both CR conditions (CRP − 1.9 ± 0.2 kg; CRC − 1.9 ± 0.1 kg, both *p* < 0.001) and in CON (− 0.8 ± 0.3 kg, *p* < 0.01), although weight loss in CR conditions was greater than in CON (*d* = 1.88, *p* < 0.01). In both CR conditions, participants lost significant fat mass (CRP − 0.5 ± 0.1 kg, *p* < 0.01; CRC − 0.6 ± 0.2 kg, *p* < 0.001) and lean mass (CRP − 1.3 ± 0.3 kg, *p* < 0.001; CRC − 1.4 ± 0.2 kg, *p* = 0.001). Changes in fat mass (− 0.2 ± 0.2 kg) and lean mass (− 0.5 ± 0.5 kg) in CON were not significant (*p* > 0.10). The differences in fat mass (*d* = 0.95, *p* = 0.05) and lean mass (*d* = 0.91, *p* = 0.06) losses between CR and CON did not achieve statistical significance. Additionally, morning urine-specific gravity did not differ between time points (*p* = 0.22).

### Growth hormone response to resistance exercise

All participants successfully performed 25 repetitions during the 5 × 5 in each condition besides one participant who performed only 24 repetitions in one condition. No characteristics of the resistance exercise bout, including warm-up volume, working set volume, total volume, proportion of working volume in total volume, or time rested differed between conditions (all *p* > 0.50) [Table S1].

**Fig. 2:**
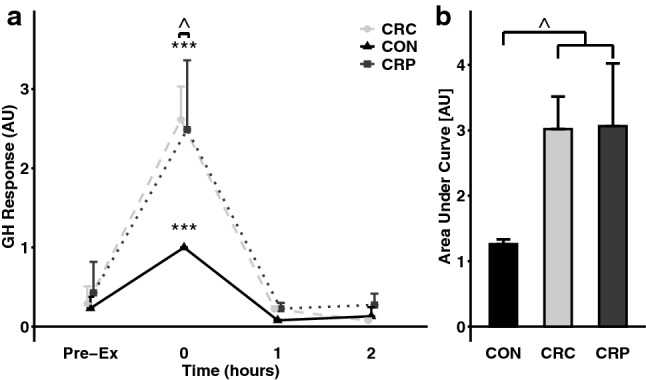
Growth hormone (GH) response to resistance exercise bout after 2 days of caloric restriction (CR) or control (CON) followed by post-exercise ingestion of protein (CRP) or carbohydrate (CRC, CON). GH concentrations are normalized to the GH peak in CON (*n* = 6). ***Indcates *p* < 0.001 vs Pre-Ex ^indicates *p* < 0.05 vs CON

In response to the exercise bout, GH was elevated immediately post-exercise in all conditions (all *p* < 0.001, 0 h vs Pre-Ex) (Fig. 2a) and returned to pre-exercise concentrations within 1 h [Table S2]. GH concentrations in CRC and CRP, respectively, peaked at 2.6 ± 0.4 and 2.5 ± 0.9 times the concentrations observed in CON. Together, peak GH concentrations during the two CR conditions were greater than peak concentrations during CON (*d* = 1.17, *p* < 0.05), resulting in a greater AUC response in the two CR conditions compared to CON (*d* = 1.20, *p* < 0.05) (Fig. 2b).

### IGF-1 response to diet and resistance exercise

**Fig. 3:**
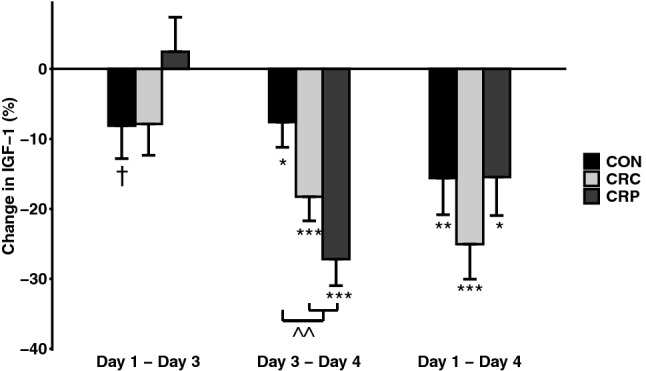
Changes in IGF1 by condition and between time points, adjusted to control for order effects (*n* = 7). †Indicates *p* < 0.10; *indicates *p* < 0.05; ** indicates *p* < 0.01; ***indicates *p* < 0.001; ^^indicates *p* < 0.01 vs CON

**Fig. 4:**
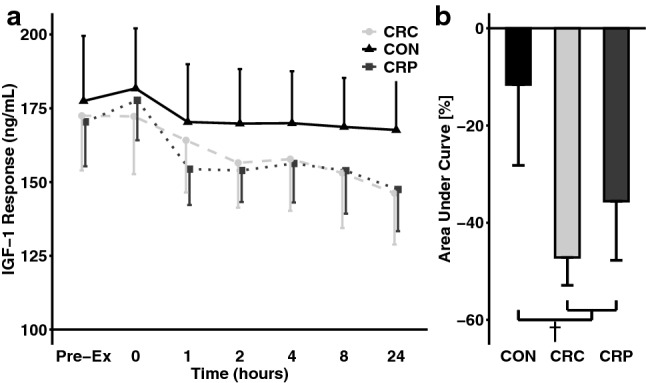
Insulin-like Growth Factor-1 (IGF-1) response to resistance exercise bout after 2 days of caloric restriction (CR) or control (CON) followed by post-exercise ingestion of protein (CRP) or carbohydrate (CRC, CON) (*n* = 7). †Indicates *p* < 0.10 vs CON

Following 2 days of controlled diet (day 1 to day 3), IGF-1 did not decrease significantly in any condition (all *p* > 0.05) (Fig. 3). However, in response to the resistance exercise bout, IGF-1 decreased in all conditions (day 3 to day 4, all *p* < 0.05). The decrease in IGF-1 was significantly greater in the CR conditions than in CON (*d* = 1.30, *p* < 0.01). Serial blood draws show the decline in AUC for IGF-1 over the 24 h following the resistance exercise bout (Fig. 4a) which was greater in the two CR conditions compared to CON (Fig. 4b), though this difference did not achieve statistical significance (*d* = 0.93, *p* = 0.06). There were no observable differences between CRC and CRP on the IGF-1 response to resistance exercise.

### Bone turnover response to diet and resistance exercise

**Fig. 5:**
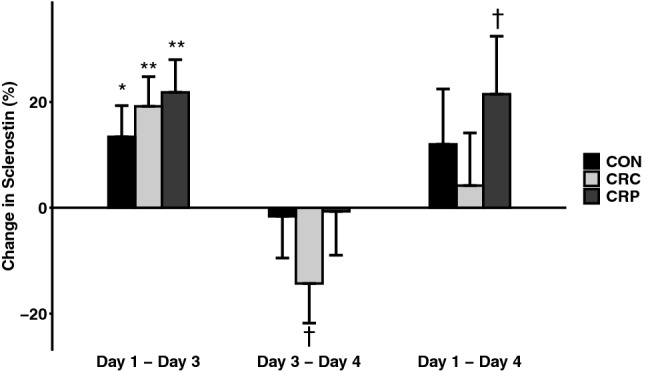
Change in sclerostin by condition and between time points, adjusted to control for order effects (*n* = 7). †Indicates *p* < 0.10; *indicates *p* < 0.05; **indicates *p* < 0.01

Sclerostin increased in all conditions following 2 days on a controlled diet (day 1 to day 3, all *p* < 0.05) (Fig. 5). However, following the resistance exercise bout, none of the observed elevations in sclerostin throughout each condition remained significant (day 1 to day 4, all *p* > 0.06).

**Fig. 6:**
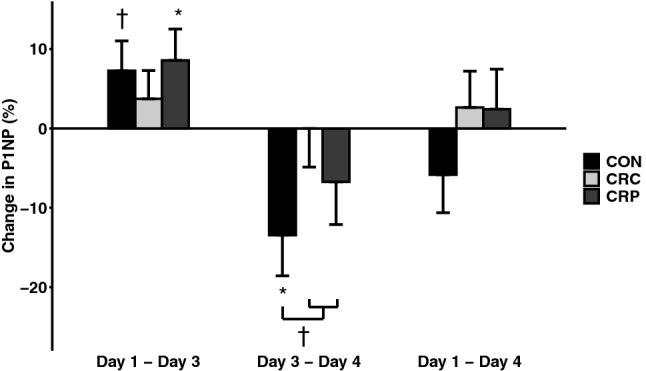
Change in P1NP by condition and between time points adjusted to control for order effects (*n* = 7). †Indicates *p* < 0.10; *indicates *p* < 0.05

P1NP increased in CRP (*p* = 0.04) and CON (*p* = 0.07) following 2 days on a controlled diet (day 1 to day 3), although the latter did not achieve statistical significance (Fig. 6). P1NP decreased 24 h after resistance exercise (day 3 to day 4) in CON (*p* = 0.02), but in neither of the CR conditions, though the difference between CON and the CR conditions did not achieve statistical significance (*d* = 0.80, *p* = 0.06). Overall, no significant changes in P1NP were observed across each condition as a whole (day 1 to day 4).

## Discussion

The primary finding of the present intervention is that 3 days of caloric restriction at an energy availability of 15 kcal kg FFM^−1^ induced considerable anabolic resistance to a heavy resistance exercise bout. This effect occurred whilst consuming 1.2 g kg BW^−1^ protein and continued in the presence of post-exercise supplementation of either protein or carbohydrate.

We are the first to quantify the GH and IGF-1 responses to a heavy resistance exercise bout during caloric restriction. Our results show reduced IGF-1 responses 24-h following a single bout of resistance exercise despite greater peak GH concentrations immediately after the bout in the calorie-restricted state compared to the control condition. This dysregulated combination has previously been observed following exposure to low-energy availability in non-exercising populations (Loucks and Thuma 2003). However, we are the first to demonstrate this anabolic resistance induced by short-term caloric restriction persists in the presence of a potent anabolic stimulus, such as resistance exercise. We speculate that this state may reduce the potential benefits of resistance exercise to muscle mass and bone in the calorie-restricted state, but further research is needed to explore strategies for restoring the sensitivity of IGF-1 to GH stimulation and test whether outcomes such as bone mineral density or lean mass retention are improved.

We also observed a significant increase in sclerostin in response to 2 days of caloric restriction at an energy availability of 15 kcal kg FFM^−1^ without exercise. We observed an increase in sclerostin of a similar magnitude after 5 days at the same energy availability, while participants performed daily aerobic exercise and consumed a low-protein (0.8 g kg bw^−1^) diet (Murphy et al. 2019). However, without exercise, we observed a similar magnitude of change in just 2 days despite a greater amount of protein (1.2 g kg bw^−1^). In agreement with the previous literature, weight-bearing exercise prevented a further increase in sclerostin (Armamento-Villareal et al. 2012). In fact, the changes observed following 2 days of controlled diet without exercise were not seen across the full 3 days of any conditions, suggesting that a single resistance exercise bout may be sufficient to attenuate the elevations in sclerostin caused by short-term caloric restriction.

The changes which we observed in bone formation, measured by P1NP, were not as consistent as those observed in sclerostin. Contrary to what we hypothesized, P1NP increased over the first 2 days in one calorie-restricted condition, as well as the control condition, and decreased following the resistance exercise bout in the control condition. Resistance training has previously been shown to increase P1NP in postmenopausal women (Pasqualini et al. 2019), but we did not observe an increase 24 h after resistance exercise in this intervention. Further research is needed to replicate and confirm the influence of resistance exercise on bone markers in the calorie-restricted state.

Post-exercise supplementation of 30 g whey protein offered no discernable benefit to any outcomes reported here compared to consuming an isocaloric amount of maltodextrin. Protein feeding has been shown to provide a quicker, greater stimulus for GH release compared to carbohydrate (Pallotta and Kennedy 1968). However, in that intervention, GH concentrations peaked 2 h following protein ingestion in the absence of an exercise stimulus. In the present intervention, GH peaked immediately following the resistance exercise bout, suggesting that the resistance exercise bout may have overshadowed any potential benefit of protein ingestion by causing GH release to enter a refractory period during the time-window protein feeding may stimulate GH release. Interestingly, pre-exercise supplementation of protein has been shown to impair the GH response to a single bout of resistance exercise (Hulmi et al. 2005). Together, these results suggest that stimulation of GH by either resistance exercise or protein may suppress the ability of the other to stimulate its release by entering a refractory period. Additional research is needed to determine the interplay of exercise and protein stimulation of endocrine anabolic factors, especially during a state of caloric restriction.

The same amount of whey protein used in this intervention has previously been reported to enhance muscle protein synthesis above resting rates observed at energy balance following 5 days of caloric restriction at an energy availability of 30 kcal kg FFM^−1^ (Areta et al. 2014). However, a recent study utilizing a unique, large exercise prescription of 45 min of one-arm cranking and 8 h of walking per day for 4 days reported that skeletal muscle became immune to the anabolic effects of whey protein during an energy deficit of 5500 kcal day^−1^ (Martin-Rincon et al. 2019). Though the energy deficit targeted in that intervention exceeds our own average by more than threefold, it is plausible that whey protein may become ineffective below a threshold of energy availability and could explain why our post-exercise protein supplementation did not appear to have an impact. Additional interventions with varying levels of energy availability are needed to establish a threshold of energy availability for the benefits of whey protein.

One point of criticism about the present intervention was the inability of our control condition to maintain weight and induce a positive post-exercise IGF-1 response in all participants. However, reported post-exercise IGF-1 responses are highly variable (Kraemer et al. 2017) and the energy availability of 40 kcal kg FFM^−1^ used in this intervention has successfully maintained weight in a previous intervention (Koehler et al. 2016). We acknowledge that different methodologies for the quantification of GH exist (Hymer et al. 2001) and the methodology employed in the present intervention may not include a comprehensive quantification of all isoforms (Hymer et al. 2019). Thus, future studies should seek to confirm our findings utilizing alternative methodologies to that in the present intervention. We further acknowledge a comprehensive quantification of the acute GH and IGF-1 responses to a bout of exercise which may benefit from additional sampling points between 0- and 1-h post-exercise; however, we observed clear differences between our control condition and the calorie-restricted conditions with the time points measured. That we still observed clear differences between the CR conditions and CON in spite of these limitations speaks to the robustness of the effects induced by our CR conditions.

The primary objective of the present intervention was to characterize the anabolic endocrine response to a bout of resistance exercise during caloric restriction. While we generally refer to this as an anabolic response, we acknowledge that there are other components of the general anabolic response, such as muscle protein synthesis (Hector et al. 2015, 2018; Murphy et al. 2015; Areta et al. 2014). However, we felt that there was a gap in the literature with regards to the endocrine response to resistance exercise under caloric restriction. While it has been questioned whether the acute IGF-1 response to exercise predicts hypertrophy during energy balance (Kraemer et al. 2017), there is evidence suggesting that signaling involved in muscle turnover downstream of IGF-1 is suppressed during caloric restriction (Martin-Rincon et al. 2019) and the suppression of IGF-1 itself during caloric restriction is linked to bone loss (De Souza and Williams 2005). This suggests that the activity of IGF-1 during caloric restriction may differ from that during energy balance. The purpose of this study was to confirm that stimulation of IGF-1 secretion by GH is impaired during caloric restriction even in the face of potent anabolic stimulation. With this framework established, subsequent studies should assess the effectiveness of countermeasures to protect against the development of anabolic resistance and, subsequently, maximize the benefits of resistance exercise in the calorie-restricted state to skeletal muscle and bone.

## Conclusion

Three days of caloric restriction to an energy availability of 15 kcal kg FFM^−1^ induced considerable anabolic resistance—characterized by increased GH secretion and reduced IGF-1 secretion—to a heavy resistance exercise bout. This response occurred in the presence of post-exercise supplementation of either protein or carbohydrate. Despite this, a bout of resistance exercise did mitigate increases in sclerostin observed during each intervention. These results suggest that while resistance exercise in the calorie-restricted state can positively influence downstream tissues, such as bone, the persistence of anabolic resistance may limit the effectiveness of resistance exercise during the calorie-restricted state. Additional measures beyond post-exercise macronutrient supplementation are necessary to enhance the sensitivity of the IGF-1:GH axis to resistance exercise during caloric restriction.

## Electronic supplementary material

Below is the link to the electronic supplementary material.

Supplementary file1 (TIF 655 kb)

Supplementary file2 (DOCX 14 kb)

## Abbreviations

AUC: Area under the curve
BW: Body weight
CON: Control condition
CR: Caloric restriction conditions
CRC: Caloric restriction with carbohydrate condition
CRP: Caloric restriction with protein condition
GH: Growth hormone
IGF-1: Insulin-like growth factor-1
P1NP: N-terminal propeptide of type-1 collagen
RIR: Repetitions-in-reserve

## References

[CR1] Areta JL, Burke LM, Camera DM, West DW, Crawshay S, Moore DR, Stellingwerff T, Phillips SM, Hawley JA, Coffey VG (2014). Reduced resting skeletal muscle protein synthesis is rescued by resistance exercise and protein ingestion following short-term energy deficit. Am J Physiol Endocrinol Metab.

[CR2] Armamento-Villareal R, Sadler C, Napoli N, Shah K, Chode S, Sinacore DR, Qualls C, Villareal DT (2012). Weight loss in obese older adults increases serum sclerostin and impairs hip geometry but both are prevented by exercise training. J Bone Miner Res.

[CR3] Armamento-Villareal R, Aguirre L, Waters DL, Napoli N, Qualls C, Villareal DT (2019). Effect of aerobic or resistance exercise, or both, on bone mineral density and bone metabolism in obese older adults while dieting: a randomized controlled trial. J Bone Miner Res.

[CR4] Armstrong LE, Pumerantz AC, Fiala KA, Roti MW, Kavouras SA, Casa DJ, Maresh CM (2010). Human hydration indices: acute and longitudinal reference values. Int J Sport Nutr Exerc Metab.

[CR5] Ballard TL, Clapper JA, Specker BL, Binkley TL, Vukovich MD (2005). Effect of protein supplementation during a 6-mo strength and conditioning program on insulin-like growth factor I and markers of bone turnover in young adults. Am J Clin Nutr.

[CR6] Beavers KM, Beavers DP, Martin SB, Marsh AP, Lyles MF, Lenchik L, Shapses SA, Nicklas BJ (2017). Change in bone mineral density during weight loss with resistance versus aerobic exercise training in older adults. J Gerontol A Biol Sci Med Sci.

[CR7] Borrud LG, Flegal KM, Looker AC, Everhart JE, Harris TB, Shepherd JA (2010). Body composition data for individuals 8 years of age and older: US population, 1999–2004. Vital Health Stat.

[CR8] Carbone JW, Pasiakos SM, Vislocky LM, Anderson JM, Rodriguez NR (2014). Effects of short-term energy deficit on muscle protein breakdown and intramuscular proteolysis in normal-weight young adults. Appl Physiol Nutr Metab.

[CR9] Clark JE (2015). Erratum to: diet, exercise or diet with exercise: comparing the effectiveness of treatment options for weight-loss and changes in fitness for adults (18–65 years old) who are overfat, or obese; systematic review and meta-analysis. J Diabetes Metab Disord.

[CR10] Cohen J (1988). Statistical power analysis for the behavioral sciences.

[CR38] De Souza MJ, Williams NI (2005). Beyond hypoestrogenism in amenorrheic athletes: energy deficiency as a contributing factor for bone loss. Curr Sports Med Rep.

[CR11] Ensrud KE, Vo TN, Burghardt AJ, Schousboe JT, Cauley JA, Taylor BC, Hoffman AR, Orwoll ES, Lane NE, Langsetmo L (2018). Weight loss in men in late life and bone strength and microarchitecture: a prospective study. Osteoporos Int.

[CR12] Fazeli PK, Klibanski A (2014). Determinants of GH resistance in malnutrition. J Endocrinol.

[CR13] Forbes GB (2000). Body fat content influences the body composition response to nutrition and exercise. Ann N Y Acad Sci.

[CR14] Fujimura R, Ashizawa N, Watanabe M, Mukai N, Amagai H, Fukubayashi T, Hayashi K, Tokuyama K, Suzuki M (1997). Effect of resistance exercise training on bone formation and resorption in young male subjects assessed by biomarkers of bone metabolism. J Bone Miner Res.

[CR15] Häkkinen K, Pakarinen A, Newton RU, Kraemer WJ (1998). Acute hormone responses to heavy resistance lower and upper extremity exercise in young versus old men. Eur J Appl Physiol Occup Physiol.

[CR16] Hector AJ, Marcotte GR, Churchward-Venne TA, Murphy CH, Breen L, von Allmen M, Baker SK, Phillips SM (2015). Whey protein supplementation preserves postprandial myofibrillar protein synthesis during short-term energy restriction in overweight and obese adults. J Nutr.

[CR17] Hector AJ, McGlory C, Damas F, Mazara N, Baker SK, Phillips SM (2018). Pronounced energy restriction with elevated protein intake results in no change in proteolysis and reductions in skeletal muscle protein synthesis that are mitigated by resistance exercise. FASEB J.

[CR18] Hulmi JJ, Volek JS, Selänne H, Mero AA (2005). Protein ingestion prior to strength exercise affects blood hormones and metabolism. Med Sci Sports Exerc.

[CR19] Hymer WC, Kraemer WJ, Nindl BC, Marx JO, Benson DE, Welsch JR, Mazzetti SA, Volek JS, Deaver DR (2001). Characteristics of circulating growth hormone in women after acute heavy resistance exercise. Am J Physiol Endocrinol Metab.

[CR20] Hymer WC, Kennett MJ, Maji SK, Gosselink KL, McCall GE, Grindeland RE, Post EM, Kraemer WJ (2019). Bioactive growth hormone in humans: controversies, complexities and concepts. Growth Horm IGF Res.

[CR21] Ihle R, Loucks AB (2004). Dose-response relationships between energy availability and bone turnover in young exercising women. J Bone Miner Res.

[CR22] Koehler K, Hoerner NR, Gibbs JC, Zinner C, Braun H, De Souza MJ, Schaenzer W (2016). Low energy availability in exercising men is associated with reduced leptin and insulin but not with changes in other metabolic hormones. J Sports Sci.

[CR23] Kraemer WJ, Ratamess NA, Nindl BC (2017). Recovery responses of testosterone, growth hormone, and IGF-1 after resistance exercise. J Appl Physiol.

[CR24] Longland TM, Oikawa SY, Mitchell CJ, Devries MC, Phillips SM (2016). Higher compared with lower dietary protein during an energy deficit combined with intense exercise promotes greater lean mass gain and fat mass loss: a randomized trial. Am J Clin Nutr.

[CR25] Loucks AB, Thuma JR (2003). Luteinizing hormone pulsatility is disrupted at a threshold of energy availability in regularly menstruating women. J Clin Endocrinol Metab.

[CR26] Luk HY, Kraemer WJ, Szivak TK, Flanagan SD, Hooper DR, Kupchak BR, Comstock BA, Dunn-Lewis C, Vingren JL, DuPont WH, Hymer WC (2015). Acute resistance exercise stimulates sex-specific dimeric immunoreactive growth hormone responses. Growth Horm IGF Res.

[CR27] Martin-Rincon M, Perez-Suarez I, Pérez-López A, Ponce-González JG, Morales-Alamo D, de Pablos-Velasco P, Holmberg HC, Calbet JAL (2019). Protein synthesis signaling in skeletal muscle is refractory to whey protein ingestion during a severe energy deficit evoked by prolonged exercise and caloric restriction. Int J Obes (Lond).

[CR28] Moore DR, Churchward-Venne TA, Witard O, Breen L, Burd NA, Tipton KD, Phillips SM (2015). Protein ingestion to stimulate myofibrillar protein synthesis requires greater relative protein intakes in healthy older versus younger men. J Gerontol A Biol Sci Med Sci.

[CR29] Murphy CH, Churchward-Venne TA, Mitchell CJ, Kolar NM, Kassis A, Karagounis LG, Burke LM, Hawley JA, Phillips SM (2015). Hypoenergetic diet-induced reductions in myofibrillar protein synthesis are restored with resistance training and balanced daily protein ingestion in older men. Am J Physiol Endocrinol Metab.

[CR30] Murphy C, Marks-Nelson E, Koehler K (2019) Increased protein intake prevents elevations in sclerostin during short-term diet- and exercise-induced weight loss. Paper presented at the Experimental Biology 2019, Orlando

[CR31] Niemann I, Hannemann A, Nauck M, Spielhagen C, Völzke H, Wallaschofski H, Friedrich N (2013). The association between insulin-like growth factor I and bone turnover markers in the general adult population. Bone.

[CR32] Pallotta JA, Kennedy PJ (1968). Response of plasma insulin and growth hormone to carbohydrate and protein feeding. Metabolism.

[CR33] Papageorgiou M, Elliott-Sale KJ, Parsons A, Tang JCY, Greeves JP, Fraser WD, Sale C (2017). Effects of reduced energy availability on bone metabolism in women and men. Bone.

[CR34] Pasiakos SM, Cao JJ, Margolis LM, Sauter ER, Whigham LD, McClung JP, Rood JC, Carbone JW, Combs GF, Young AJ (2013). Effects of high-protein diets on fat-free mass and muscle protein synthesis following weight loss: a randomized controlled trial. FASEB J.

[CR35] Pasqualini L, Ministrini S, Lombardini R, Bagaglia F, Paltriccia R, Pippi R, Collebrusco L, Reginato E, Sbroma Tomaro E, Marini E, D'Abbondanza M, Scarponi AM, De Feo P, Pirro M (2019). Effects of a 3-month weight-bearing and resistance exercise training on circulating osteogenic cells and bone formation markers in postmenopausal women with low bone mass. Osteoporos Int.

[CR36] Rubin MR, Kraemer WJ, Maresh CM, Volek JS, Ratamess NA, Vanheest JL, Silvestre R, French DN, Sharman MJ, Judelson DA, Gómez AL, Vescovi JD, Hymer WC (2005). High-affinity growth hormone binding protein and acute heavy resistance exercise. Med Sci Sports Exerc.

[CR37] Sardeli AV, Komatsu TR, Mori MA, Gáspari AF, Chacon-Mikahil MPT (2018). Resistance training prevents muscle loss induced by caloric restriction in obese elderly individuals: a systematic review and meta-analysis. Nutrients.

[CR39] Thomas GA, Kraemer WJ, Kennett MJ, Comstock BA, Maresh CM, Denegar CR, Volek JS (2011). Immunoreactive and bioactive growth hormone responses to resistance exercise in men who are lean or obese. J Appl Physiol 1985.

[CR40] Tritos NA, Klibanski A (2016). Effects of growth hormone on bone. Prog Mol Biol Transl Sci.

[CR41] Villalon KL, Gozansky WS, Van Pelt RE, Wolfe P, Jankowski CM, Schwartz RS, Kohrt WM (2011). A losing battle: weight regain does not restore weight loss-induced bone loss in postmenopausal women. Obesity (Silver Spring).

[CR42] Villareal DT, Fontana L, Weiss EP, Racette SB, Steger-May K, Schechtman KB, Klein S, Holloszy JO (2006). Bone mineral density response to caloric restriction-induced weight loss or exercise-induced weight loss: a randomized controlled trial. Arch Intern Med.

[CR43] Villareal DT, Fontana L, Das SK, Redman L, Smith SR, Saltzman E, Bales C, Rochon J, Pieper C, Huang M, Lewis M, Schwartz AV (2016). Effect of two-year caloric restriction on bone metabolism and bone mineral density in non-obese younger adults: a randomized clinical trial. J Bone Miner Res.

[CR44] Villareal DT, Aguirre L, Gurney AB, Waters DL, Sinacore DR, Colombo E, Armamento-Villareal R, Qualls C (2017). Aerobic or resistance exercise, or both, in dieting obese older adults. N Engl J Med.

[CR45] Vottero A, Guzzetti C, Loche S (2013). New aspects of the physiology of the GH-IGF-1 axis. Endocr Dev.

[CR46] Wahl P, Mathes S, Köhler K, Achtzehn S, Bloch W, Mester J (2013). Acute metabolic, hormonal, and psychological responses to different endurance training protocols. Horm Metab Res.

[CR47] Weinheimer EM, Sands LP, Campbell WW (2010). A systematic review of the separate and combined effects of energy restriction and exercise on fat-free mass in middle-aged and older adults: implications for sarcopenic obesity. Nutr Rev.

[CR48] Weiss EP, Jordan RC, Frese EM, Albert SG, Villareal DT (2017). Effects of weight loss on lean mass, strength, bone, and aerobic capacity. Med Sci Sports Exerc.

[CR49] Yang YJ, Martin BR, Boushey CJ (2010). Development and evaluation of a brief calcium assessment tool for adolescents. J Am Diet Assoc.

