# Correction to: Caloric restriction induces anabolic resistance to resistance exercise

**DOI:** 10.1007/s00421-021-04750-0

**Published:** 2021-06-28

**Authors:** Chaise Murphy, Karsten Koehler

**Affiliations:** 1 Department of Nutrition and Health Sciences, University of Nebraska-Lincoln, Lincoln, NE USA; 2 Department of Sport and Health Sciences, Technical University of Munich, Uptown München-Campus D, Georg-Brauchle-Ring 60/62, 80992 München, Germany

## Correction to: European Journal of Applied Physiology (2020) 120:1155–1164 10.1007/s00421-020-04354-0

The article Caloric restriction induces anabolic resistance to resistance exercise, written by Chaise Murphy and Karsten Koehler, was originally published Online First without Open Access. After publication in volume 120, issue 5, pages 1155–1164 the author decided to opt for Open Choice and to make the article an Open Access publication. Therefore, the copyright of the article has been changed to © The Author(s) 2021 and this article is licensed under a Creative Commons Attribution 4.0 International License, which permits use, sharing, adaptation, distribution and reproduction in any medium or format, as long as you give appropriate credit to the original author(s) and the source, provide a link to the Creative Commons licence, and indicate if changes were made. The images or other third party material in this article are included in the article’s Creative Commons licence, unless indicated otherwise in a credit line to the material. If material is not included in the article’s Creative Commons licence and your intended use is not permitted by statutory regulation or exceeds the permitted use, you will need to obtain permission directly from the copyright holder. To view a copy of this licence, visit http://creativecommons.org/licenses/by/4.0/.

The original article has been corrected.

