# Supplementary figures and images for: Caloric restriction induces anabolic resistance to resistance exercise
Source: Eur J Appl Physiol. 2020 Mar 31;120(5):1155–64. doi: 10.1007/s00421-020-04354-0 (PMC8233264; doi:10.1007/s00421-020-04354-0)

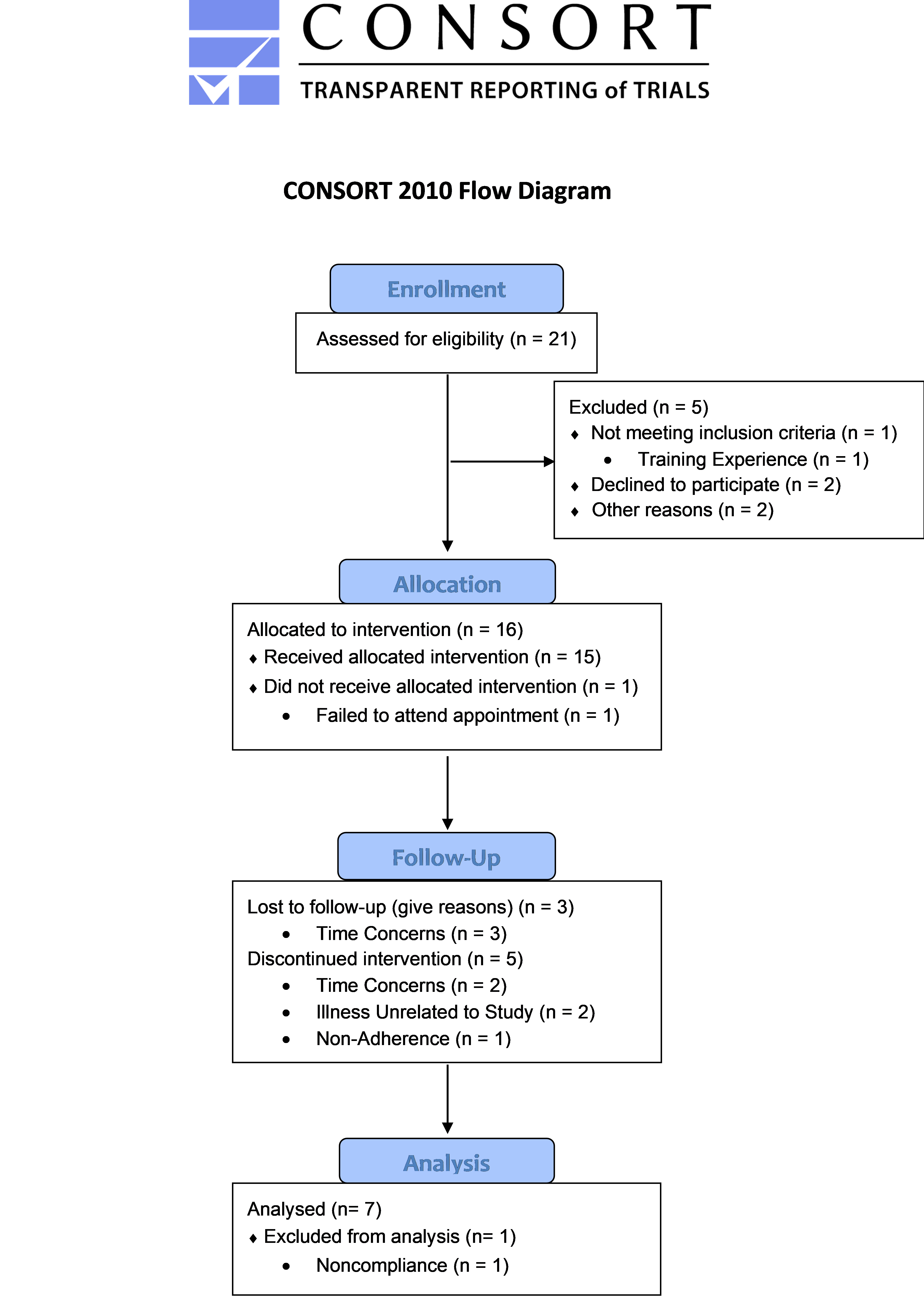

Supplement: Supplementary file 1 — Supplementary file1 (TIF 655 kb) [file 421_2020_4354_MOESM1_ESM.tif]
