# Supplementary material for: Caloric restriction induces anabolic resistance to resistance exercise
Source: Eur J Appl Physiol. 2020 Mar 31;120(5):1155–64. doi: 10.1007/s00421-020-04354-0 (PMC8233264; doi:10.1007/s00421-020-04354-0)
Supplement: Supplementary file 2 — Supplementary file2 (DOCX 14 kb) [file 421_2020_4354_MOESM2_ESM.docx]

**Figure S1.** CONSORT Flow Diagram for present study.

**Tables**

| **Condition** | **CRP** | **CRC** | **CON** |
| --- | --- | --- | --- |
| **Warm-Up Volume**  **(kg· kg bw^-1^ ·reps)** | 12.6 ± 2.8 | 12.6 ± 1.6 | 12.3 ± 2.5 |
| **Working Volume**  **(kg· kg bw^-1^ ·reps)** | 35.1 ± 2.3 | 34.6 ± 2.3 | 35.2 ± 2.2 |
| **Total Volume**  **(kg· kg bw^-1^ ·reps)** | 47.8 ± 4.6 | 47.2 ± 3.8 | 47.4 ± 4.0 |
| **Working / Total**  **Volume** | 75.0 ± 3.4% | 73.8 ± 1.4% | 75.2 ± 3.4% |
| **Rest Time**  **(Average)** | 3:45 ± 0:20 | 3:27 ± 0:17 | 3:42 ± 0:14 |

**Supplementary Table 1.** Characteristics of exercise bouts by condition (n =7).

| **Condition** | **Pre-Ex** | **0** | **1** | **2** |
| --- | --- | --- | --- | --- |
| **CON** | 936 ± 525 | 4914 ± 1663 | 383 ± 131 | 186 ± 96 |
| **CRC** | 1373 ± 829 | 12619 ± 4093 | 960 ± 263 | 576 ± 400 |
| **CRP** | 1305 ± 1152 | 9179 ± 3047 | 889 ± 308 | 1234 ± 663 |

**Supplementary Table 2.** Mean Growth Hormone concentrations (pg/mL) by condition and time point (n = 6).
